# Circulating microRNAs in prostate cancer—Non-invasive biomarkers for diagnosis, prognosis, and therapy: A review

**DOI:** 10.17305/bb.2025.12971

**Published:** 2025-10-06

**Authors:** Ema Volar, Borna Vuković, Ivan Franin, Zrinka Madunić, Anita Bijelić, Ivana Čelap, Nino Sinčić, Igor Tomašković, Jure Murgić, Monika Ulamec

**Affiliations:** 1 University of Zagreb, School of Medicine, Zagreb, Croatia; 2 Clinical Department of Pathology and Cytology “Ljudevit Jurak”, Sestre Milosrdnice University Hospital Center, Zagreb, Croatia; 3 Oncology and Nuclear Medicine Department, Sestre Milosrdnice University Hospital Centre, Zagreb, Croatia; 4 AstraZeneca, Zagreb, Croatia; 5 Department of Biology, Josip Juraj Strossmayer University of Osijek, Osijek, Croatia; 6 Clinical Department of Chemistry, Sestre Milosrdnice University Hospital Centre, Zagreb, Croatia; 7 Faculty of Pharmacy and Biochemistry, University of Zagreb, Zagreb, Croatia; 8 Department of Medical Biology, School of Medicine, University of Zagreb, Zagreb, Croatia; 9 Scientific Center of Excellence for Reproductive and Regenerative Medicine, University of Zagreb, School of Medicine, Zagreb, Croatia; 10 Clinical Department of Urology, Sestre Milosrdnice University Hospital Centre, Zagreb, Croatia; 11 School of Medicine, Catholic University of Croatia, Zagreb, Croatia; 12 Pathology Department, School of Medicine University of Zagreb, Zagreb, Croatia

**Keywords:** Prostate cancer, miRNA, biomarkers, diagnosis, therapy, tumor microenvironment

## Abstract

Prostate cancer (PC) is a common malignancy driven by interacting genetic, environmental, and lifestyle factors, including hereditary mutations (*BRCA1/2, HPC1, AR* variants), premalignant lesions [proliferative inflammatory atrophy (PIA), prostatic intraepithelial neoplasia (PIN)], and Western dietary patterns. This narrative review aims to synthesize evidence on the role of microRNAs (miRNAs) in PC pathogenesis and clinical management across diagnosis, prognosis, therapy, and recurrence prediction. We searched PubMed/MEDLINE (2004–present) using predefined terms, screened reference lists, excluded outdated records, and prioritized biomarker studies with AUC ≥ 0.85. Current diagnostic pathways—digital rectal examination, prostate-specific antigen (PSA) testing, multiparametric MRI, and Gleason-based International Society of Urological Pathology (ISUP) grading—are complemented by molecular tools (4Kscore, PHI, SelectMDx, TMPRSS2–ERG, PCA3, ConfirmMDx). MiRNAs, key post-transcriptional regulators, contribute to PC via dysregulated biogenesis and modulation of androgen receptor (AR) signaling within an inflamed, remodeled tumor microenvironment. Circulating and exosomal miRNAs (notably miR-21, miR-375, and miR-182-5p) exhibit greater specificity and stability than PSA, enabling non-invasive diagnosis, risk stratification, treatment monitoring, and recurrence prediction. Therapeutic approaches—antagomirs, sponges, miRNA masks, and CRISPR editing—show preclinical promise, while chemical modifications [peptide nucleic acids (PNAs), locked nucleic acids (LNAs), C2′ modifications] improve stability and delivery but remain limited by biodistribution, tissue penetration, off-target effects, and immunogenicity. In conclusion, standardized workflows and multicenter validation, integrated with clinical and imaging data, are essential to translate miRNA-based tools into precision PC management.

## Introduction

### Prostate cancer

Prostate cancer (PC) is one of the most prevalent malignancies affecting men worldwide, with approximately one in eight men diagnosed during their lifetime. It is responsible for mortality in roughly one out of every 44 diagnosed cases. Global estimates indicate that around 1,276,106 new cases of PC are identified annually, leading to approximately 358,989 deaths each year. These figures are anticipated to rise due to demographic shifts such as population aging, increased life expectancy, and advancements in diagnostics and treatment options. By 2030, the global burden of PC is projected to reach 1.7 million new cases, with an estimated 499,000 associated deaths.

PC significantly impacts health-related quality of life, particularly among middle-aged and older men. Pathological changes in the prostate are most commonly observed after age 40 and are often accompanied by a range of urinary and sexual symptoms. These symptoms may include increased urinary frequency, dysuria (painful urination), urinary incontinence, hematuria (blood in the urine), hematospermia (blood in the semen), nocturia, and erectile dysfunction. However, these clinical manifestations are not exclusive to PC and may also result from other prostatic conditions, such as benign prostatic hyperplasia (BPH) and various forms of prostatitis, including acute and chronic bacterial prostatitis, chronic pelvic pain syndrome, and asymptomatic inflammatory prostatitis [1, 2].

### MicroRNAs (miRNAs)

miRNAs are an evolutionarily conserved class of small, non-coding RNA molecules, typically around 22 nucleotides in length, that serve as critical post-transcriptional regulators of gene expression [3]. They are primarily transcribed by RNA polymerase II, although a subset may be transcribed by RNA polymerase III, reflecting the diversity of their regulatory control. Approximately half of miRNAs are intragenic, arising predominantly from intronic regions of protein-coding genes, while a smaller fraction originates from exonic regions. The remaining miRNAs are intergenic and are transcribed independently under the control of their own promoter elements.

The biogenesis of miRNAs occurs through two primary pathways: the canonical and non-canonical pathways. In the canonical pathway, primary miRNA transcripts (pri-miRNAs) are processed in the nucleus by the microprocessor complex, which comprises the RNase III enzyme Drosha and the RNA-binding protein (RBP) DGCR8. This complex recognizes conserved motifs (e.g., GGAC) to generate precursor miRNAs (pre-miRNAs) with a characteristic 2-nucleotide 3′ overhang [3]. Pre-miRNAs are exported to the cytoplasm via the Exportin-5/Ran-GTP complex and are further processed by Dicer to yield a ∼22-nucleotide miRNA duplex. One strand, known as the guide strand, is incorporated into the RNA-induced silencing complex (RISC) with Argonaute (AGO) proteins, while the passenger strand is typically degraded.

Non-canonical pathways bypass one or more steps of this classical route. Drosha-independent pathways generate miRNAs called mirtrons through mRNA splicing and Exportin-1-mediated export, while Dicer-independent pathways utilize AGO2 to directly process pre-miRNAs, circumventing Dicer cleavage [4].

MiRNAs exhibit remarkable stability in various biological fluids—including plasma, serum, urine, saliva, synovial fluid, and bile—due to their association with AGO proteins, extracellular vesicles, and high-density lipoproteins. This stability positions them as promising non-invasive biomarkers for disease diagnosis, prognosis, and therapeutic monitoring across cancer, cardiovascular, and inflammatory diseases [5]. Functionally, miRNAs regulate gene expression by binding to microRNA-responsive elements (MREs) in the 3′-untranslated regions of target mRNAs. Depending on complementarity, the RISC complex can induce mRNA cleavage or inhibit translation, thereby modulating cell proliferation, differentiation, and development [6]. The activity of miRNAs is highly context-dependent; the same miRNA may function as an oncogene (oncomir) or tumor suppressor (oncosuppressor) depending on tissue type, target genes, and signaling networks. MiRNAs also mediate cellular responses to stressors, including hypoxia, nutrient deprivation, and DNA damage. Compared to small interfering RNAs (siRNAs), miRNAs provide finely tuned regulation of gene networks, enabling precise adjustment of gene expression in diverse tissues under physiological or pathological conditions [6].

## Materials and methods

For this narrative review, we explored the existing literature using PubMed/MEDLINE and examined key references from major journals and publishers. Search terms included “prostate carcinoma,” “miRNA,” “PSA,” “tumor microenvironment,” “biomarkers in prostate carcinoma,” “prostate cancer therapy,” and “miRNA therapy.” We focused on studies published after 2004, excluding those with outdated records. For miRNA biomarker studies, only those reporting an area under the curve (AUC) of 0.85 or higher were included [7].

## Pathogenesis and etiologic factors in PC

### A multifactorial disease

PC is a highly heterogeneous malignancy shaped by a dynamic interplay of genetic, environmental, and racial factors. Among the most influential determinants of risk is a positive family history, underscoring the role of hereditary predisposition in the disease’s onset and progression. Genes most implicated in PC pathogenesis include those involved in AR signaling and testosterone metabolism. These pathways are essential for the embryonic development of the prostate epithelium and play a central role in tumorigenesis and disease progression later in life [8]. Beyond androgen-related mechanisms, at least seven genetic loci have been linked to increased susceptibility to PC. Notably, the hereditary PC 1 (*HPC1*) gene, located on chromosome 1q24-25 (Table 1), which encodes ribonuclease L (RNase L), a key enzyme in the innate immune response and the interferon-alpha (IFNα) signaling pathway. RNase L is crucial for antiviral defense and apoptosis regulation. Mutations in RNase L can compromise immune surveillance, increase vulnerability to retroviral infections, and contribute to chronic prostatic inflammation, an important driver of malignant transformation in prostatic tissue [3, 9].

**Table 1:** Genes involved in the development of PC

**Gene locus**	**Mutated genes**	**Protein/enzyme encoded by the genome**	**Role in the development of cancer**
1q24-25	*HPC1* gene	Ribonuclease L (RNASEL)	Virus defence and apoptosis regulation
17p11	*HPC2/ELAC2* gene	ELAC2 protein	Regulation of proliferation by activation of the TGF- β signaling pathway
8p22	*MSR1*	Macrophage receptor 1	Unknown
17q21	*BRCA1*	Breast cancer type 1 susceptibility protein	A more aggressive form of PC
13q13	*BRCA2*	Breast cancer type 2 susceptibility protein	Increased risk of developing PC
16p12	*PALB2*	Partner and localizer of BRCA2 protein	Familial PC
Xq26.3-q27.3	*AR*	Androgen receptor	Sporadic and hereditary PC

Abbreviations: AR: Androgen receptor; BRCA1: Breast cancer type 1 susceptibility protein; BRCA2: Breast cancer type 2 susceptibility protein; ELAC2: ELAC2 protein; HPC1: Hereditary prostate cancer 1; HPC2: Hereditary prostate cancer 2; MSR1: Macrophage scavenger receptor 1; PALB2: Partner and localizer of BRCA2; PC: Prostate cancer; RNASEL: Ribonuclease L; TGF-β: Transforming growth factor beta.

### Genetic susceptibility and DNA repair pathways in PC

Another significant gene is *HPC2/ELAC2*, located on chromosome 17p11, which encodes the enzyme zinc phosphodiesterase ELAC2. This enzyme is involved in the post-transcriptional processing of *SMAD2* (Mothers against decapentaplegic homolog 2), thereby modulating cell proliferation through the activation of the transforming growth factor-beta (TGF-β) signaling cascade. Similarly, the macrophage scavenger receptor 1 (*MSR1*) gene, positioned on chromosome 8p22, has been implicated in susceptibility to PC; however, its precise contribution to tumorigenesis remains poorly understood [10, 11].

In addition to these loci, germline mutations in *BRCA1* and *BRCA2*—genes associated with hereditary breast and ovarian cancers—have also been identified in men with PC. Notably, *BRCA2* mutations confer a significantly elevated risk for developing aggressive and early-onset forms of the disease. Furthermore, the *PALB2* (partner and localizer of *BRCA2*) gene which encodes a protein integral to BRCA2 function, has been implicated in familial PC cases [3, 10].

The X chromosome also contributes to hereditary risk, particularly through variants in the androgen receptor (*AR*) gene [12]. Structural alterations, including deletions in the Xq26.3–q27.3 region, have been observed in both familial and sporadic PC cases, further emphasizing the importance of androgen signaling in the disease’s pathogenesis [8].

### Environmental and lifestyle risk factors

Beyond genetic predisposition, dietary and lifestyle factors significantly influence the incidence and progression of PC. The disease is notably more prevalent in highly industrialized nations, where dietary habits are characterized by elevated intake of saturated fats, total caloric content, and processed meats. Such nutritional patterns are thought to promote cancer progression through multiple mechanisms, including enhanced androgen activity, increased oxidative stress, and the biosynthesis of lipid-derived pro-inflammatory mediators, such as leukotrienes and prostaglandins. These biochemical processes may collectively stimulate basal metabolic activity and create a microenvironment conducive to tumor initiation and progression. Consequently, PC incidence is higher in Western populations compared to those in Asian and African regions [13], highlighting the potential impact of modifiable lifestyle factors on disease burden [3].

### Morphology and early lesions in prostate carcinogenesis

Proliferative inflammatory atrophy (PIA) and prostatic intraepithelial neoplasia (PIN) represent key premalignant histopathological alterations implicated in the early stages of prostate carcinogenesis. PIA is characterized by regions of epithelial atrophy accompanied by chronic inflammation and increased epithelial cell proliferation. It is hypothesized to arise in response to oxidative stress, infection, or hormonal imbalances, creating a pro-tumorigenic microenvironment through the generation of reactive oxygen species, cytokines, and inflammatory mediators. Over time, PIA lesions may evolve into high-grade PIN (HGPIN), a well-established precursor to invasive prostatic adenocarcinoma [10].

HGPIN is defined by architectural and cytological atypia within prostatic ducts and acini, including nuclear enlargement, nucleolar prominence, and stratification of luminal cells, while preserving the basal cell layer. Molecular studies have revealed that PIN and PC often share genetic and epigenetic alterations—such as chromosomal instability, *TMPRSS2-ERG* gene fusion, and loss of *PTEN*—supporting the concept of a progressive neoplastic continuum. Recognition and monitoring of these premalignant lesions are essential, as they provide valuable insight into disease pathogenesis and may serve as targets for early detection and chemopreventive strategies [9].

### miRNAs: Biogenesis, function, and role in PC

Overexpression of certain miRNAs has been observed in a wide range of cancers, including PC. This dysregulation often results from chromosomal mutations, excessive miRNA synthesis, or disturbances in epigenetic regulation [6]. A large-scale genomic study by Calin et al. demonstrated that cancer-associated miRNA genes are specifically distributed across chromosomes, with many located in genomic regions frequently mutated in tumor cells, referred to as cancer-associated genomic regions (CAGRs). CAGRs can be classified into several types: tumor suppressor genes, where mutations in both alleles are necessary for cancer development; oncogenes, where a mutation in just one allele is sufficient to trigger tumor formation; breakpoint regions; and fragile sites (FRAs), which facilitate chromosomal recombination, translocation, or integration of plasmids or viral DNA [14]. Excessive miRNA synthesis can lead to errors in the maturation of pri-miRNA [3]. Emerging evidence highlights the pivotal role of dysregulated miRNAs in the pathogenesis and progression of PC. Aberrations in miRNA expression profiles can disrupt normal cellular homeostasis by suppressing tumor suppressor genes or enhancing oncogenic signaling pathways. Several miRNAs have been identified as direct regulators of AR signaling, either by regulating its expression, binding to its transcript, or modulating the activity of AR-associated coregulators. These findings highlight the dual role of miRNAs as both diagnostic and prognostic biomarkers, as well as promising therapeutic targets in the management of PC [3].

### Tumor microenvironment (TME)

The TME plays a crucial role in the pathobiology of advanced PC. It encompasses a dynamic and heterogeneous milieu composed of extracellular matrix (ECM) components, neural elements, vasculature, immune infiltrates, mesenchymal-derived stromal cells, and other specialized cellular constituents. These elements interact through intricate networks involving chemokines, cytokines, growth factors, and matrix-remodeling enzymes, with bidirectional communication between tumor and stromal cells predominantly mediated through autocrine and paracrine signaling pathways.

Preclinical models have demonstrated that early oncogenic events in PC are tightly coupled with stromal remodeling within the TME. Hallmark changes include increased fibroblast proliferation, neovascularization, apoptotic resistance, and the phenotypic transition of myofibroblasts into cancer-associated fibroblasts (CAFs). In this context, myofibroblasts—typically activated during tissue repair—acquire a tumor-promoting phenotype, similar to the response observed in wound healing. Epithelial-derived TGF-β plays a pivotal role in circumventing myofibroblast apoptosis, establishing a self-sustaining autocrine feedback loop that facilitates persistent TGF-β secretion, enhanced survival signaling, and metabolic reprogramming. This milieu fosters the release of oncogenic mediators such as circulating miRNAs and interleukins, further promoting the differentiation of myofibroblasts into CAFs. The resultant desmoplastic stroma, rich in newly generated CAFs, perpetuates tumor progression through a continuous cycle of stromal activation [15].

Advanced PC, particularly cases exhibiting higher Gleason scores, is frequently associated with a stroma infiltrated by immune effector cells. This immunological infiltration is thought to arise from sustained prostatic tissue irritation caused by factors such as chronic urinary tract infections, intraprostatic urinary reflux, high-fat dietary intake, and elevated estrogenic stimuli. Consequently, there is increased recruitment of CD3+ T lymphocytes, tumor-associated macrophages, and mast cells into the tumor-adjacent stroma. These immune populations contribute to a chronic inflammatory state characterized by elevated levels of pro-inflammatory cytokines and chemokines, including tumor necrosis factor-alpha (TNF-α) and activation of the nuclear factor kappa B (NF-κB) pathway. These mediators regulate multiple oncogenic processes, such as angiogenesis, cellular proliferation, and immune evasion, which are instrumental in PC progression [10].

Ultimately, the reciprocal crosstalk between malignant epithelial cells and the surrounding stroma drives profound molecular and phenotypic changes within the TME. These alterations modulate tumor aggressiveness, metastatic potential, and therapeutic resistance, underscoring the significance of the stromal compartment as a critical determinant of PC outcomes [10, 15].

## Diagnostic and prognostic algorithms

Digital rectal examination (DRE) is a crucial element of the initial clinical evaluation for suspected prostatic pathology. Palpable abnormalities, such as glandular enlargement, induration, or nodular irregularities, may indicate the presence of an underlying neoplastic process [9].

### Prostate-specific antigen (PSA) testing: Use and limitations

Following DRE, serum PSA levels are routinely measured. PSA, a glycoprotein secreted by prostatic epithelium, serves as a widely recognized biomarker for prostate health. Although elevated PSA levels can suggest malignancy, they are not specific to cancer and may also indicate benign conditions such as BPH or prostatitis. PSA values exceeding 4 µg/L are generally considered suspicious, with concentrations between 4–10 µg/L correlating with an estimated 25% risk of PC, and levels above 10 µg/L indicating approximately a 50% risk of malignancy [10].

### Challenges of PSA screening and the need for novel biomarkers

Despite its extensive use, PSA testing suffers from limited specificity and sensitivity, particularly in differentiating indolent tumors from clinically significant PCs. Consequently, alternative biomarkers that could enhance diagnostic and prognostic capabilities are under active investigation. The limitations of PSA testing lead to numerous unnecessary biopsies, which are invasive and carry potential complications, including hematuria, urinary retention, and infectious sequelae such as urosepsis and prostatitis. To improve diagnostic accuracy, multiparametric magnetic resonance imaging (mpMRI) is increasingly utilized to guide targeted biopsies and facilitate histological confirmation [16].

### Histopathological diagnosis and tumor grading

Histopathological evaluations predominantly reveal prostatic adenocarcinoma, which originates from glandular epithelium. Tumor grading is conducted using the Gleason scoring system, categorizing PCs based on architectural differentiation. In 2016, the International Society of Urogenital Pathology (ISUP) introduced a revised grading system:

- ISUP group 1: Gleason ≤6 (3+3); well-formed glands.
- ISUP group 2: Gleason 3+4═7; predominantly well-formed glands with a smaller proportion of poorly differentiated ones.
- ISUP group 3: Gleason 4+3═7; mostly poorly differentiated glands.
- ISUP group 4: Gleason 8 (4+4, 3+5, 5+3); significant architectural atypia.
- ISUP group 5: Gleason 9–10; absence of glandular structure, frequent necrosis, high aggressiveness.

This classification improves clinical applicability, enhancing risk stratification and informing treatment decisions [17, 18].

### Biomarkers in PC: Diagnostic and prognostic utility

Biomarkers are essential in the clinical management of PC, playing critical roles in diagnosis, disease staging, risk stratification, and therapeutic decision-making. While PSA remains the cornerstone of initial PC screening, its limited specificity has driven the exploration and integration of additional molecular and biochemical markers to enhance diagnostic accuracy and minimize overtreatment.

Among these, the four-kallikrein (4K) panel has gained prominence in clinical practice, particularly for men with elevated PSA levels. This plasma-based assay quantifies four kallikrein isoforms—total PSA (tPSA), free PSA (fPSA), intact PSA, and human kallikrein 2 (hK2)—and integrates them into a predictive algorithm to estimate the likelihood of high-grade PC. The 4K score has proven effective in reducing unnecessary biopsies and improving the detection of clinically significant disease [19].

The Prostate Health Index (PHI) is another validated serum-based biomarker that combines tPSA, fPSA, and the [-2]proPSA (p2PSA) isoform into a single numerical value. PHI has demonstrated improved specificity over PSA alone, particularly in the diagnostic “grey zone” (PSA 2–10 ng/mL), potentially reducing unnecessary biopsies by up to 40% [20].

Urine-based biomarker assays also provide non-invasive alternatives to traditional screening methods. SelectMDx, performed after DRE to enrich prostate-derived RNA in urine, evaluates mRNA expression of *HOXC6, DLX1*, and *KLK3* in conjunction with PSA density (PSAD). This test stratifies the risk of high-grade PC and has shown the potential to avoid up to 53% of unnecessary biopsies [21].

The *TMPRSS2–ERG* gene fusion, a molecular alteration found in approximately 50% of PC cases, can be detected in post-DRE urine samples. This fusion, involving the androgen-regulated transmembrane protease serine 2 (*TMPRSS2*) and the ETS-related gene (*ERG*), is associated with tumor aggressiveness and is under investigation as a prognostic marker and a tool for monitoring response to androgen deprivation therapy (ADT) [22].

PC Antigen 3 (PCA3), a prostate-specific non-coding RNA overexpressed in more than 95% of primary PCs, represents another urinary biomarker with enhanced specificity compared to PSA. The PCA3 score, derived from the ratio of PCA3 to PSA mRNA in urine, may assist in guiding biopsy decisions and reducing unnecessary procedures [23].

ConfirmMDx is a tissue-based epigenetic assay used post-biopsy to assess DNA hypermethylation in genes associated with tumorigenesis, including *GSTP1, APC,* and *RASSF1*. This test is particularly valuable for patients with histologically negative biopsies but ongoing clinical suspicion of PC, as it detects field effects suggestive of occult malignancy. ConfirmMDx has demonstrated improved diagnostic accuracy, reduced repeat biopsy rates, and identified patients at increased risk for clinically significant disease [13].

Collectively, these biomarkers, when used alongside PSA and clinical parameters, enhance diagnostic precision, optimize patient selection for prostate biopsy, and provide critical prognostic insights, particularly in the context of personalized medicine in PC care.

### Circulating miRNA as biomarker in PC

Circulating miRNAs in plasma have emerged as promising non-invasive biomarkers for diagnosing and prognosticating PC [5]. Unlike messenger RNAs, miRNAs demonstrate exceptional physicochemical stability in body fluids, including plasma, serum, and seminal fluid. They are inherently resistant to endogenous RNases, extreme pH shifts, temperature fluctuations, and repeated freeze-thaw cycles, facilitating reliable detection even under suboptimal storage conditions.

The origin and stability of circulating miRNAs can be explained by several mechanisms.

1. **Passive release:** miRNAs can enter circulation through cell lysis during apoptosis, necrosis, or tumor-associated cellular turnover, reflecting the intracellular miRNA of damaged or malignant cells.
2. **Active secretion via extracellular vesicles (EVs):** Prostate cells actively secrete miRNAs within exosomes (30–150 nm) and microvesicles. These vesicles facilitate intercellular communication, influence TME remodeling, angiogenesis, and immune evasion, and exhibit remarkable stability in circulation. Notably, prostate-derived EVs can enter urine, enabling non-invasive detection [24].
3. **Non-vesicular secretion:** miRNAs may also be released independently of vesicles, often complexed with RBPs such as Argonaute 2 (AGO2) and Nucleophosmin 1 (NPM1), which protect them from enzymatic degradation. The AGO2–miRNA complex accounts for over 90% of plasma miRNAs, underscoring the significance of non-vesicular transport [25].

**Table 2:** miRNA panels used for the diagnosis and prediction of PC treatment

**Panel**	***miRNA***	**Purpose**
Moya et al.	miR-98-5p, miR152-3p, miR-326 and miR-4289	Isolation of patients who have PC
Liu et al.	miR-223, miR-24 and miR-375	Distinguishing indolent from progressive disease course
Brase et al.	miR-141 and miR- 375	Predictors of tumour metastatic potential
Shen et al.	miR-20a, miR-21, miR-145, and miR-221	Distinguishing high-risk and low-risk PC
Hoey et al.	miR-20a, miR-17, miR-20b and miR-106a	Distinguishing high-risk and low-risk PC
Biddara et al.	miR-182-5p and miR-375-3p	Prediction of advanced PC
Alhasan et al.	miR-106a, miR-135a, miR-200c, miR-433, and miR-605	Prediction of high-risk PC
Abramovic et al.	miR-375-3p, miR-182-5p	Distinguishing PC from BPH, comparison of miRNA and PSA as biomarkers

Abbreviations: PC: Prostate cancer; BPH: Benign prostatic hyperplasia; PSA: Prostate-specific antigen; miRNA: MicroRNA; miR: Mature microRNA; 5p/3p: Derived from the 5-prime or 3-prime arm of the pre-miRNA.

A subset of miRNAs, termed oncomiRs, are upregulated in tumors and promote proliferation, invasion, angiogenesis, and metastasis. For instance, miR-21 is consistently overexpressed in prostate and other cancers, with elevated serum levels correlating with disease presence and progression. Advances in high-throughput miRNA profiling, including microRNAome sequencing, qRT-PCR, and antisense-based detection, have improved the sensitivity and specificity of miRNA-based diagnostics [26].

1. **Clinical utility in PC:** Numerous studies have identified circulating miRNAs with potential as diagnostic and prognostic biomarkers for PC.
2. **Diagnostic panels:** Moya et al. reported a four-miRNA panel—miR-98-5p, miR-152-3p, miR-326, and miR-4289—that distinguished PC patients from healthy controls with an AUC of 0.88 [27]. Liu et al. proposed a panel of miR-223, miR-24, and miR-375 to differentiate indolent from progressive disease (AUC = 0.690) [28].
3. **Prognostic biomarkers:** Brase et al. identified miR-141 and miR-375 as markers associated with metastatic PC [29]. Shen et al. demonstrated that a serum panel including miR-20a, miR-21, miR-145, and miR-221 could discriminate high-risk from low-risk disease (AUC ═ 0.824) [30]. Wood and Brown highlighted miR-218-5p as a predictor of bone metastases (AUC = 0.86) [31]. Hoey et al. developed a panel of miR-20a, miR-17, miR-20b, and miR-106a predictive of high-risk post-prostatectomy patients [32]. Biddara et al. showed that miR-182-5p and miR-375-3p were associated with advanced disease stages [33]. Alhasan et al. proposed a five-miRNA signature (miR-106a, miR-135a, miR-200c, miR-433, and miR-605) capable of distinguishing aggressive from indolent PC with 89% accuracy [34] (Table 2).
4. **Therapeutic monitoring:** Certain miRNAs correlate with treatment response. miR-21 has been linked to enhanced radiosensitivity, while miR-146a and miR-155 are associated with post-radiotherapy inflammatory responses. Abramovic et al. quantified miR-375-3p, miR-182-5p, miR-21-5p, and miR-148a-3p in blood and seminal plasma from PC and BPH patients, revealing that miR-182-5p and miR-375-3p were significantly overexpressed in PC, achieving a combined specificity of 90.2%, markedly outperforming PSA (specificity = 25% at 4 µg/L) [10, 35].

In summary, these findings underscore the potential of circulating and exosomal miRNAs as robust, minimally invasive biomarkers for early detection, risk stratification, prognosis, and therapeutic monitoring in PC. However, translating these findings into clinical practice necessitates standardized isolation protocols, larger multi-center validation studies, and systematic evaluation of prostate-specific miRNAs.

## Therapeutic challenges and future directions

While miRNA-based therapies have demonstrated potential in tumor treatment, several challenges impede their clinical application. Unmodified miRNAs are rapidly degraded by serum nucleases and excreted by the kidneys, resulting in a limited serum half-life. Furthermore, they may activate the immune system, leading to immunotoxicity. A primary challenge lies in the effective delivery of miRNAs to tumor tissues, ensuring sufficient tissue penetration while minimizing adverse effects. Chemical modifications—such as peptide nucleic acids (PNAs), locked nucleic acids (LNAs), and C2’ modifications—can enhance resistance to degradation, reduce immunotoxicity, and improve binding to target tissues. Nevertheless, the development of both viral and non-viral miRNA carriers remains essential. While viral vectors offer high delivery efficiency, they often provoke immunotoxic responses. Conversely, non-viral carriers are generally safer but less efficient [36, 37].

### miRNA therapies

In PC therapy, the inhibition of oncogenic miRNAs can be effectively achieved using synthetic anti-miRNA oligonucleotides (AMOs), typically comprising 17–22 nucleotides that anneal to complementary miRNA sequences. This binding disrupts the RISC, preventing the miRNA from targeting tumor suppressor transcripts, thereby reducing tumor proliferation and metastatic potential. A notable subclass of chemically engineered AMOs, known as antagomirs, has shown *in vivo* efficacy in silencing miRNAs in PC models. For instance, direct intratumoral administration of antagomirs targeting miR-221 and miR-222 resulted in downregulation of these oncogenic miRNAs and reactivation of tumor suppressor pathways, significantly impeding tumor growth in murine subcutaneous xenograft models [38].

Innovative approaches in AMO design include the use of crosslinked 2′-O-methyl (2′-OMe) RNA duplexes incorporating 2-amino-6-vinylpurine (AVP), which achieve selective interstrand crosslinking with uracil residues. A recent study developed such crosslinked duplexes targeting miR-21, demonstrating superior inhibitory activity compared to commercially available LNA-modified inhibitors, suggesting enhanced target affinity and stability [39]. Further optimization of AMO constructs through the incorporation of LNA or PNA modifications has been explored, particularly against miR-21. LNA-modified anti-miR-21 oligonucleotides have been shown to suppress PC cell viability and tumor burden in xenograft models. Similarly, systemic administration of PNA-based anti-miR-21 significantly reduced bone metastases *in vivo* [40].

Beyond direct miRNA antagonism, alternative strategies include miRNA sponges—vector-encoded RNA transcripts containing multiple tandem miRNA response elements. These constructs sequester miRNAs, thereby diminishing their regulatory activity on tumor suppressor gene targets [37]. Another strategy involves miRNA masks—antisense oligonucleotides designed to hybridize with miRNA binding sites on target mRNAs rather than with the miRNAs themselves. This approach sterically hinders RISC binding and allows normal translation of tumor suppressor genes, circumventing oncogenic miRNA interference [41]. Additionally, genome editing technologies such as CRISPR/Cas9 are being investigated for their potential to delete or mutate oncogenic miRNA loci, offering a durable method to silence miRNAs implicated in PC progression and metastasis [3].

## Conclusion

PC remains a significant global health concern due to its high incidence, heterogeneous progression, and limited therapeutic efficacy in a substantial subset of patients. The integration of molecular biomarkers, particularly miRNAs, holds considerable promise for enhancing early detection, risk stratification, and personalized therapy. Emerging evidence underscores the dual utility of circulating and exosomal miRNAs as non-invasive diagnostic and prognostic tools, as well as potential therapeutic agents capable of modulating oncogenic pathways. However, several limitations hinder the clinical translation of miRNA-based applications. Preanalytical variability—including differences in sample collection, processing, storage, and miRNA isolation methods—contributes to inconsistencies in assay performance and comparability across studies. Moreover, many studies are limited by small cohort sizes, retrospective designs, or single-center analyses, constraining their findings. Variability in miRNA expression due to patient heterogeneity, comorbidities, or concurrent medications further complicates interpretation.

To address these challenges, standardization of detection platforms and protocols is essential, alongside rigorous validation across diverse populations. Furthermore, the development of safe and effective miRNA delivery systems, including chemical modifications, nanocarrier technologies, and genome-editing approaches, is crucial for realizing the therapeutic potential of miRNAs.

Future research should focus on large-scale, multi-center clinical trials, integrated biomarker panels, and longitudinal studies to assess the predictive and therapeutic utility of miRNAs in PC. Interdisciplinary collaboration among molecular biologists, clinicians, and bioengineers will be vital for translating these insights into clinically actionable strategies, ultimately facilitating more precise, less invasive, and more effective management of PC.

## Graphical abstract

**Figure:**
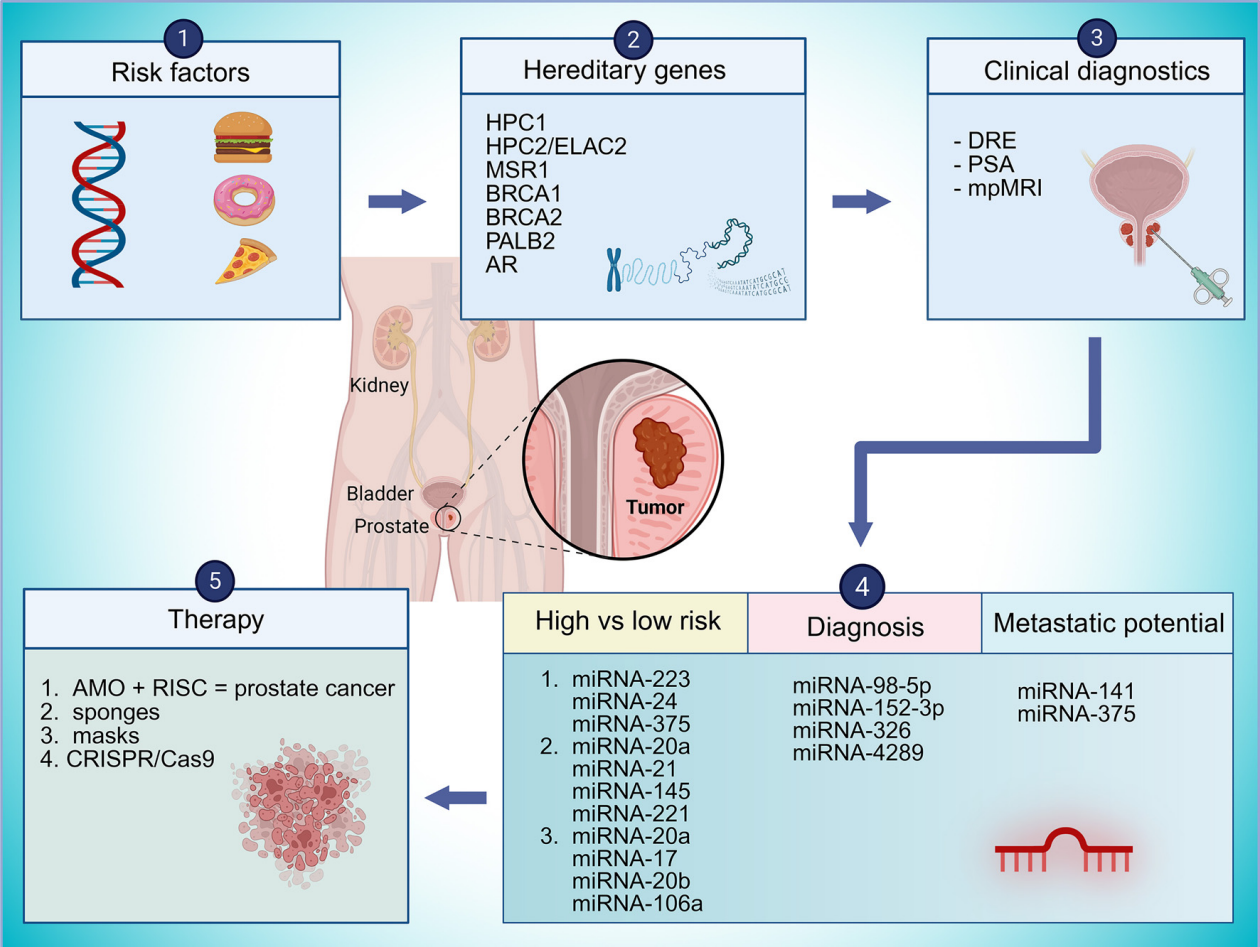


## Data Availability

The authors declare that full data is public and available.
